# Perception of vibrotactile distance on the back

**DOI:** 10.1038/s41598-020-74835-x

**Published:** 2020-10-21

**Authors:** Myrthe A. Plaisier, Lotte I. N. Sap, Astrid M. L. Kappers

**Affiliations:** 1 Dynamics and Control, Department of Mechanical Engineering, Eindhoven University of Technology, Eindhoven, The Netherlands; 2 Human Technology Interaction, Department of Industrial Engineering and Innovation Sciences, Eindhoven University of Technology, Eindhoven, The Netherlands; 3 Control Systems Technology, Department of Mechanical Engineering, Eindhoven University of Technology, Eindhoven, The Netherlands

**Keywords:** Perception, Human behaviour

## Abstract

Vibrotactile displays worn on the back can be used as sensory substitution device. Often vibrotactile stimulation is chosen because vibration motors are easy to incorporate and relatively cheap. When designing such displays knowledge about vibrotactile perception on the back is crucial. In the current study we investigated distance perception. Biases in distance perception can explain spatial distortions that occur when, for instance, tracing a shape using vibration. We investigated the effect of orientation (horizontal vs vertical), the effect of positioning with respect to the spine and the effect of switching vibration motors on sequentially versus simultaneously. Our study includes four conditions. The condition which had a horizontal orientation with both vibration motors switching on sequentially on the same side of the spine was chosen is the baseline condition. The other three conditions were compared to this baseline condition. We found that distances felt longer in the vertical direction than in the horizontal direction. Furthermore, distances were perceived to be longer when vibration motors were distributed on both sides of the spine compared to when they were on the same side. Finally, distances felt shorter when vibration motors were switched on simultaneously compared to sequentially. In the simultaneous case a distance of 4 cm was not clearly perceived differently than a distance of 12 cm. When designing vibrotactile displays these anisotropies in perceived distance need to be taken into account because otherwise the intended shape will not match the perceived shape. Also, dynamically presented distances are more clearly perceived than static distances. This finding supports recommendations made in previous studies that dynamic patterns are easier to perceive than static patterns.

## Introduction

Sensory substitution devices are often designed to help compensate for vision or hearing loss. Especially when there is a combination of vision and hearing loss the tactile modality is used to compensate. Braille displays are a well known example using the tactile modality to compensate the loss of vision. In that case tactile information is displayed to the finger tips. This is an obvious choice since the finger tips have a very high spatial acuity compared to other body parts. Despite their high spatial acuity, the finger tips are not in all cases the best choice. The hands can be occupied with a different task and are therefore not always available for communication. Another consideration is the available surface area. For instance, when the information that needs to be conveyed is represented by a shape that is traced, a body part with a larger surface area can be more desirable. Available surface area is one of the main reasons that devices have been designed to display information to the back of a person. The back has a relatively low spatial acuity^1^, but it can still be a desirable location for sensory subsitution. For instance, it provides a large surface area which can be preferable despite low spatial acuity. Kristjánsson and colleagues have also argued that passive body parts might be preferable for sensory substitution as it enables devices to be, for instance, hands-free^2^. In addition, it has been shown that pattern recognition is actually better on the torso than on the forearm^3^.

In the current study we will focus on vibrotactile stimulation on the back. Vibrotactile stimulation is often chosen over other types of stimulation because it is easy and cheap to implement using vibration motors. Previous studies have shown that such displays have been used to, for instance, convey shapes and letters^4–6^, but also military hand signals^7^. Vibration patterns can be displayed statically where a number of vibration motors is switched on and off at the same time or dynamically where motors are switched on and off sequentially. The second case is most similar to tracing a shape on the back. It has been shown that for shapes and letters dynamic patterns are easier to recognise than static ones^4–6,8^. So, the recognisability of vibrotactile patterns is determined by both spatial and temporal aspects.

Spatial distortions of, for instance, a perceived shape might be related to variations in spatial acuity. It has been shown that there are anisotropies in terms of discrimination threshold and localisation accuracy on the trunk. Vibrotactile stimulation has been shown to be more accurately localised near the spine and navel than at other positions around the torso^9,10^, although reduced sensitivity has been found directly on the spine^11^. Furthermore, Hoffmann and colleagues have shown that vibrations on the back were more accurately localised with respect to one another in the horizontal direction than in the vertical direction^11^. In the same study it was also found that tactile sensitivity near the spine was higher than further towards the side, but only in the horizontal direction.

Anisotropy in spatial acuity does not necessarily have to lead to traced shapes being perceived as distorted. For pressure stimulation there are, however, many examples of distortions in distance perception which would lead to distorted shape perception. Longo and Haggard showed that when presenting a line on the hand of a participant by pressing two rods onto the skin, the length of this line was perceived to be different depending on whether it was presented along or across the hand^12^. This effect of distance being perceived as longer when presented across body width compared to along body length has been found for other body parts as well such as the forearm, thigh, shin and face^13–15^. There are variations across body locations in the size of this effect. For instance, this effect is larger for the hairy skin on the dorsal side of the hand than on the palm^16^, and it was not found for the belly^17^.

These biases in distance perception have been linked to variations in spatial acuity. Distances on body parts with high spatial acuity tend to be perceived as longer than on body parts with lower spatial acuity. This is known as Weber’s illusion^18^. Longo and Haggard have introduced a model based on receptive fields^12^. They argued that to estimate the distance between two locations that are stimulated, the number of unstimulated receptive fields in between is used. The receptive field density is higher for areas with higher spatial acuity and there will be many unstimulated receptive fields in between two stimulation points. Also, receptive fields are often oval shaped and elongated along the distal axis. This can explain the perceived difference between distances across the body width compared to along the body length for pressure stimulation. It has been argued though that differences in receptive field density cannot be the whole explanation. Taylor-Clarke et al. have argued that based on differences in spatial acuity the differences in perceived length would be expected to be much larger than observed^19^. Also, they found that tactile distance perception was altered after viewing the hand via size-enhanced video.

Another reason for biases in distance perception to occur is the presence of anchor points^20^. Anchor points are usually joints. Spatial localisation can be better near such reference points than further away. For instance, localisation of a vibrotactile stimulus on the forearm has been shown to be best near the wrist or elbow^21^. The spine might also be considered to be an anchor point even though it does not move in the way that joints do. Tactile localisation has been shown to be better near the spine compared to other locations on the back^9,11^. The spine is on the body mid-line and its bilateral cortical representation might also play a role^9^.

There is some evidence that anisotropy in terms of distance perception also exists on the back for vibrotactile stimulation. Kappers and colleagues recently found that perception of directionality is anisotropic across the back^22^. Furthermore, Novich and Eagleman found that a line traced using vibration motors along the diagonal was relatively often confused with a horizontal line^8^. Gaining understanding of perceptual biases in distance perception on the back will make it possible to anticipate distortions of shapes or letters drawn on the back.

In the current study we set out to systematically investigate distance perception on the back using vibrotactile stimulation. We use horizontal distances displayed on one side of the spine as our baseline condition. We compare this to distances presented in the vertical direction to test if indeed distances are systematically perceived differently between the two orientations. To test how the spine influences distance perception we compared the horizontal baseline condition to a horizontal distance presented with a vibration motor on the left and right sides of the spine. In all these conditions two vibration motors were switched on sequentially and thus distances were presented dynamically. To compare distance perception between dynamic and static distance presentation we added also a condition that was identical to the horizontal baseline condition except that both vibration motors were turned on simultaneously. The perceived distance was measured using the method of free magnitude estimation^23^ and participants were asked to rate the size of the distance on an arbitrary scale that they were free to choose.

## Results

**Figure 1:**
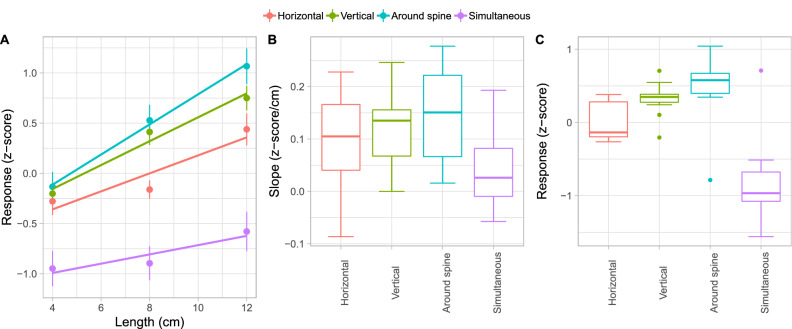
Results. (**A**) The perceptual distance ratings as a function of the presented distance. Dots indicate the mean and error bars the SE across participants. The lines indicate linear regression to the averaged distance ratings. (**B**) Boxplots of the slopes for linear regression to the individual participant’s data and (**C**) the average distance ratings for each condition. Thick lines indicate the median and the boxes indicate the 25–75% intervals. The whiskers indicate the smallest value within the 25% minus 1.5 times the inter-quartile range and the largest value between 75% plus 1.5 times the inter-quartile range. Dots indicate values outside the aforementioned ranges.

The perceptual ratings were converted to Z scores to be able to compare across participants. For each distance the average perceptual rating was calculated for each condition (Fig. 1A). It can be seen that for most conditions the slope appears to be positive which is consistent with participants using larger ratings for larger distances. To investigate this further we performed linear regression on these data from each participant individually. The $R^{2}$ values averaged over participants were 0.8, 0.7, 0.8, and 0.6 for the “Horizontal”, “Vertical”, “Around spine”, and “Simultaneous” conditions, respectively. A boxplot of the resulting slopes is shown in Fig. 1B. For each condition we used a t test on the individual participants’ slopes to test whether they differed from zero and report Bonferroni corrected values to control the type I error. The slopes were significantly larger than zero for the “Around spine” ($t(11)=6.0, p=0.0004, d=1.7$) and “Vertical” ($t(11)=5.4, p=0.0009, d=1.5$) conditions. For the “Horizontal” condition the slope was marginally significant ($t(11)=3.0, p=0.051, d= 0.9$). The slope for the “Simultaneous” condition was not significantly different from zero ($t(11)=1.9, p=0.3, d=0.6$). This indicates that in the “Simultaneous” condition the differences in distance were hardly perceived even though the largest distance was three times larger than the smallest distance.

To investigate the perceived distance further we calculated the average rating a participant gave in each condition. In Fig. 1C it can be clearly be seen that most participants rated distances to be smaller in the “Simultaneous” condition than in the other conditions. To compare between the four conditions we performed a Friedman test. We used a non-parametric test because a Shapiro–Wilks test showed a violation of the normality assumption. The Friedman test showed a significant effect ($$\chi ^{2}(3) = 22.7,p<0.0001, W=0.6$$). We followed this up with Wilcoxon signed rank tests to compare each condition against the “Horizontal” condition. We applied Bonferroni correction to the p levels reported here. This analysis showed that distance was perceived to be significantly smaller in the “Horizontal” condition than in the “Around spine” ($$p = 0.02 $$) and “Vertical” conditions ($$p = 0.02$$). Furthermore, the distance in the “Horizontal” condition was perceived to be significantly longer than in the “Simultaneous” condition ($$p = 0.03$$).

## Discussion

Our results show that the orientation in which a distance is presented on the back, the location and temporal aspects of the stimulation all systematically influence the perceived distance between two points of vibrotactile stimulation. We found that vertical distances were perceived to be significantly larger than horizontal distances when both points of stimulation were located on the same side of the spine. This is different from previous studies reporting that distances across the body width are generally perceived to be longer than along the body length when using pressure stimuli^12–16^. When using vibrotactile stimulation different receptors are activated than when pressure stimulation is used. However, the explanation proposed by Longo and Haggard in terms of receptive field density in general, which is related to spatial acuity, might also apply to vibrotactile stimulation^12^. There are studies reporting enhanced spatial acuity for vibrotactile stimulation near the spine^9,10^. Hoffmann and colleagues, however, have found a decrease in sensitivity directly on the spine^11^. This discrepancy might actually be due to stimulation directly on the spine compared to in the area directly next to the spine. Hoffmann and colleagues have argued for such an explanation as the spread of vibration is largely influenced by the underlying tissue^11^. Directly on the spine there is not much fleshy tissue. In the same study Hoffmann and colleagues also reported that spatial acuity in the horizontal direction was higher than in the vertical direction^11^. Based on the receptive field theory of Longo and Haggard^12^, our finding of vertical distances being perceived to be longer than horizontal, indicate that the vertical acuity in the area where we presented the vertical distances was higher than the horizontal acuity in the area of the more peripherally presented horizontal distances. Since we presented the vertical distances near the spine, but explicitly not directly on the spine, this is not unlikely. Our finding that distances were perceived to be larger when vibration motors were placed on the left and right sides of the spine compared to when both vibration motors were placed on the same side of the spine seems also in agreement with increased spatial acuity near the spine. However, when the motors were on the left and right sides of the spine they could also have been more easily distinguishable due to hemispheric separation^9^.

Finally, when both motors were turned on simultaneously we did not find a significant slope for the perceived distance as a function of the presented distance. This implies that a distance of 4 cm was not clearly distinguishable from a distance of 12 cm. Various studies on vibrotactile spatial acuity on the back have estimated the two-point-threshold between 13 and 60 mm depending on the vibration motor type, sequential or simultaneous presentation, the exact location of stimulation, and the psychophysical method used^10,11,24–28^. Our largest distance of 12 cm was thus twice as large as the largest estimate of the two-point-threshold. This means that participants were probably able to perceive that there were two separate points stimulated, but this of course does not mean that they could make a good estimate of the distance between these two points. Our results suggest that distance perception was much more imprecise for simultaneous stimulation than for sequential stimulation. This is in agreement with a previous study by Van Erp in which spatial localisation of vibrotactile stimuli was found to improve with increasing stimulus onset asynchronicity^10^. If stimuli can be better localised with respect to one another we would expect distances to feel longer based on Weber’s illusion. This is in agreement with our results as we found that for simultaneous stimulation distances felt shorter than for sequential stimulation. A similar effects have been reported for pressure stimuli for which the distances of simultaneously applied stimuli were perceived as closer together than sequentially applied stimuli^29^. For pressure stimuli that were presented simultaneously and relatively closely together it has been reported that the illusion can occur that a single point in between the two sites of stimulation was pressed^30^.

In fact, the absence of a significant slope for the perceived distance as a function of distance between vibration motors in the “Horizontal simultaneous” condition suggests that participants might have perceived this stimulus as single point of vibration. This is actually a known tactile illusion that can occur for vibrotactile sitmulation^31^. Two simultaneously presented vibrotactile stimuli can be perceived as a single vibration in between the two sites of stimulation. This effect can be exploited for drawing trajectories that are perceived as smoother when vibration motors are switched on sequentially with a small temporal overlap between the two. The tactile brush is an example of an algorithm which exploits this perceptual illusion^32^. The illusion is also used to increase the precision for navigational displays that convey directionality using vibration^33^.

Overall, our results have important implications for the design of vibrotactile displays worn on the back. Based on our results we can make some recommendations. First, our results suggest that dynamic patterns are easier to perceive than static patterns as distances between vibrations were poorly perceived in the static case. This is in agreement with previous studies that have found that dynamically presented patterns and shapes are easier to perceive than static ones^4,6–8,34^. Secondly, presented shapes will feel distorted due to anisotropies in perceived distance for different locations on the back. These anisotropies in distance perception need to be compensated for in order for the perceived shape to feel as the intended shape. Specifically, distances close to the spine and those that cross the spine will be perceived to be longer than those further away or not crossing the spine.

## Methods

**Figure 2:**
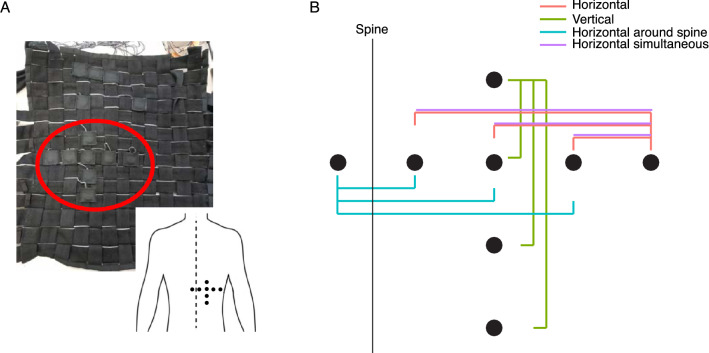
Experimental set-up and design. (**A**) A picture of the inside of the vest with the motors inserted in the pockets. The inset shows where the motors were located on the back of the participant while the vest was being worn. (**B**) Schematic representation of the configuration of the motors. The dots indicate the motors and the lines are used to indicate which motors turned on for the three distances (4, 8 and 12 cm) used.

### Participants

Twelve students of Eindhoven University of Technology participated in this experiment (4 male, 8 female). Two participants were self-reported left handed and the other ten were right handed. Their ages ranged from 19 to 23 years. Participants were asked to wear thin clothing like a t-shirt and not a thick sweater during the experiment. They received financial compensation for their participation and gave written informed consent prior to the start of the experiment. All participants were naive as to the purpose of the experiment. This study was approved by the ethical committee of the Human Technology Interaction group at Eindhoven University of Technology and the study was performed according to the local guidelines and regulations. A power analysis was performed to determine the necessary number of participants. This analysis was also included application for ethical approval.

### Experimental set-up and stimuli

Coin-style eccentric rotating mass (ERM) vibration motors were used to deliver the vibrotactile stimuli (Adafruit mini motor disc). These motors were incorporated in a vest which had small pockets in which the vibration motors could be placed (Fig. 2A). This vest was custom designed at University of Borås as part of the European project SUITCEYES. The vest was especially designed to allow for the placement of the motor to be easily adjustable and had many straps to allow tightening of the motors to the skin. The experimenter assisted the participants with putting on the vest. The motor in the center of the cross was located about 3–4 cm to the right of the T10 and T11 vertebral spinous processes. Care was taken that the motors for the “Around spine” condition were indeed positioned on the left and right sides of the spine. The experimenter also made sure that the motors for the horizontal conditions were indeed aligned horizontally and those for the vertical condition were aligned vertically. Due to variations in trunk size between participants there was some variation on the exact placement of the motors on the back. Furthermore, we asked participants to lean with the back against the backrest of the chair on which they were seated. This ensured that all motors pressed onto the skin of the participant throughout the experiment.

**Figure 3:**
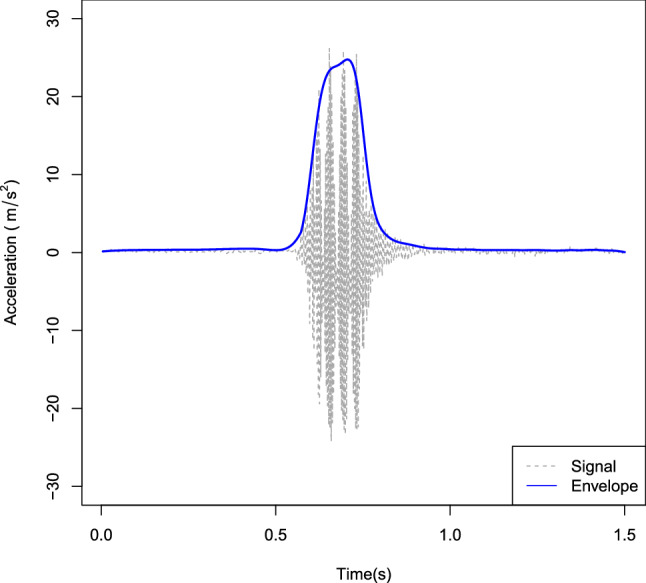
Accelerations measured when the vibration motor was switched on for 200 ms. The grey dotted line indicates the measured signal and the blue line represents the waveform envelope.

The vibration motors were provided with a voltage of 5 V using a power bank. Whether a motor received power or not was controlled using an Arduino Nano. In the “Horizontal simultaneous” condition power was supplied to both motors at the same time. In the other conditions the power to the second motor was supplied as soon as the first one was switched off. ERM motors have a a ramp-on and a ramp-off time. The ramp-on time is due to the eccentric mass having to gain momentum and the ramp-off time is because when the current is switched off the mass gradually slows down. We experimentally determined the ramp-on and ramp-off time of the type of ERM used here with the motors fixed in the same way as they were during the experiment. We performed this measurement for the motor positioned in the center of the cross by switching it on for 200 ms and measuring the accelerations using an accelerometer (Adafruit ADLX345) sampling at 460 Hz. A Hilbert transform was used to determine the waveform envelope and a Butterworth filter with a cut-off frequency of 10 Hz was subsequently used to smooth the envelope (see Fig. 3). The ramp-on time was determined as the time between the envelope raising above 5% of the peak value until 95% of the peak value was reached. The ramp-off time was of course determined as the time between the envelope dipping below 95% of the peak value and the moment at which it was below 5% of the peak value. Using this procedure the ramp-on time was determined to be 105 ms and the ramp-off time was 136 ms. This means that in the conditions where motors were switched on sequentially, there was overlap in the activity of the motors due to the ramp-off time of the first motor.

### Experimental design and task

A trial consisted of two motors turning on, either sequentially or simultaneously, and participants had to give an estimate of the distance between the two motors. The distance between the motors was 4 cm, 8 cm, or 12 cm. Motors were switched on for 200 ms. Participants were asked to rate the distance using the method of free magnitude estimation^23^. This means that they were instructed that they just had to choose an arbitrary number for the first stimulus and continue from there. They were also instructed that larger numbers should correspond with larger distances.

There were four conditions. In the “Horizontal condition” two motors that were horizontally spaced were switched on sequentially. The most rightward motor switched on first. Both motors were always on the same side of the spine. The “Vertical condition” was the same as the “Horizontal condition” except that motors were spaced vertically and the motor and the highest location switched on first. In the “Around spine condition” the motors were horizontally spaced apart and switched on sequentially, but the motors were always on opposite sides of the spine. Finally, the “Simultaneous condition” was the same as the “Horizontal condition” except that the motors were switched on simultaneously. See Fig. 2B for an overview of the spatial layout used in the different conditions. Conditions were presented randomly interleaved in a blocked random order. This means that all distances for all conditions were presented randomly interleaved. A total of ten of these randomized blocks was presented without breaks between the blocks. This method ensured that conditions were homogeneously distributed over the whole experimental session. The participants were not aware of the ordering of the trials and no breaks were introduced between blocks. Furthermore, participants wore a pair of headphones playing white noise during the experiment to mask the sound from the vibration motors. Prior to starting the experiment a block of practice trials was performed to familiarise participants with the task. The practice block consisted of all trial types in random order and therefore contained 12 trials.
